# Serum microRNA-based prediction of responsiveness to eribulin in metastatic breast cancer

**DOI:** 10.1371/journal.pone.0222024

**Published:** 2019-09-04

**Authors:** Natsuko Satomi-Tsushita, Akihiko Shimomura, Juntaro Matsuzaki, Yusuke Yamamoto, Junpei Kawauchi, Satoko Takizawa, Yoshiaki Aoki, Hiromi Sakamoto, Ken Kato, Chikako Shimizu, Takahiro Ochiya, Kenji Tamura

**Affiliations:** 1 Department of Breast and Medical Oncology, National Cancer Center Hospital, Tokyo, Japan; 2 Department of Breast Medical Oncology, National Center for Global Health and Medicine, Tokyo, Japan; 3 Division of Molecular and Cellular Medicine, National Cancer Center Research Institute, Tokyo, Japan; 4 Toray Industries, Inc., Kanagawa, Japan; 5 Dynacom Co., Ltd., Chiba, Japan; 6 Department of Biobank and Tissue Resources, National Cancer Center Research Institute, Tokyo, Japan; 7 Department of Gastrointestinal Medical Oncology, National Cancer Center Hospital, Tokyo, Japan; University of Kentucky, UNITED STATES

## Abstract

The identification of biomarkers for predicting the responsiveness to eribulin in patients with metastatic breast cancer pretreated with an anthracycline and a taxane remains an unmet need. Here, we established a serum microRNA (miRNA)-based prediction model for the emergence of new distant metastases after eribulin treatment. Serum samples were collected from metastatic breast cancer patients prior to eribulin treatment and comprehensively evaluated by miRNA microarray. The prediction model for estimating eribulin efficacy was established using the logistic LASSO regression model. Serum samples were collected from 147 patients, of which 52 developed at least one new distant metastasis after eribulin monotherapy and 95 did not develop new distant metastases. A combination of eight serum miRNAs (miR-4483, miR-8089, miR-4755-3p, miR-296-3p, miR-575, miR-4710, miR-5698 and miR-3160-5p) predicted the appearance of new distant metastases with an area under the curve of 0.79, sensitivity of 0.69 and specificity of 0.82. The serum levels of miR-8089 and miR-5698 were significantly associated with overall survival after the initiation of eribulin treatment. The present study provides evidence that serum miRNA profiling may serve as a biomarker for the responsiveness to eribulin and for predicting the development of new distant metastases in metastatic breast cancer.

## Introduction

Breast cancer was reported as the most common cancer in 2013 [1] and the fifth leading cause of cancer death in 2016 [2] among Japanese women. Although the 5 year overall survival rate among breast cancer patients exceeds 90%, the treatment of recurrent or metastatic disease remains challenging [2]. The eribulin monotherapy versus treatment of physician’s choice in patients with metastatic breast cancer (EMBRACE) trial showed that eribulin, a non-taxane microtubule inhibitor, improves overall survival in metastatic breast cancer patients pretreated with anthracycline and taxane [3]. Eribulin has been approved by the Food and Drug Administration in the United States and by the Pharmaceuticals and Medical Devices Agency in Japan since 2010, and is widely used as one of the standards of care for metastatic breast cancer in the clinical setting. However, the EMBRACE trial reported that the objective response rate of eribulin was 12%, and the clinical benefit rate was 23% [3]. Given this low response rate and high frequency of myelotoxicity and neurotoxicity, a predictive biomarker of responsiveness is critical for the use of eribulin. Despite the identification of tissue miRNA-based biomarkers for the response to eribulin in advanced soft tissue sarcoma [4], a blood-based test would increase the convenience and simplicity of diagnosis, and would therefore be more feasible for predicting the efficacy of eribulin in clinical practice.

MicroRNAs (miRNAs) are a class of small non-coding RNAs of 17–25 nucleotides. miRNA expression levels in breast cancer tissues predict the efficacy of neoadjuvant chemotherapy [5]. In addition, miRNAs remain stable in the circulation [6], and different miRNA profiles reflect various disease conditions [7]. The detection of blood plasma and serum miRNAs shows potential for the early diagnosis of cancer and for predicting prognosis and response to therapy [8]. We previously reported the diagnostic performance of the combination of five miRNAs (miR-1246, miR-1307-3p, miR-4634, miR-6861-5p and miR-6875-5p) with a sensitivity of 97.3% and specificity of 82.9% for detecting breast cancer accurately [9].

Currently, the choice of drug largely depends on the physician’s preference. A report examining predictive factors of eribulin efficacy in breast cancer showed that eribulin tended to be more effective in patients with HER2-negative, hormone receptor-negative, and triple-negative breast cancers in a pooled analysis of two phase III trials^10,11^. Here, we performed comprehensive miRNA profiling using serum samples of metastatic breast cancer patients prior to eribulin treatment. We identified a serum miRNA combination as a prognostic biomarker in metastatic breast cancer that predicted the responsiveness to eribulin to precisely select which patients would be more likely to benefit from eribulin treatment.

## Materials and methods

### Clinical samples

Patients with advanced or metastatic breast cancer who previously received anthracycline and taxane were treated with eribulin. Serum samples were collected from patients with recurrent or metastatic breast cancer ≥20 years of age who began eribulin monotherapy at National Cancer Center Hospital (NCCH) between January 2011 and December 2015. Patients who did not receive anthracycline and/or taxane, patients who lacked imaging tests to assess new distant metastases before initiating next treatments, and those lost to follow-up or dead were excluded. Before the initiation of eribulin monotherapy and after discontinuation of prior treatments, 147 serum samples were obtained from patients who were treated with eribulin after anthracycline or taxane.

### miRNA expression analysis by microarray

Total RNA was extracted and normalized using a 3D-Gene^®^ miRNA Labeling kit and a 3D-Gene^®^ Human miRNA Oligo Chip (Toray Industries, Inc.), as described in detail previously [9, 10]. All microarray data in the present study are in agreement with the Minimum Information About a Microarray Experiment guidelines and are publicly available through the GEO database (GSE110651, http://www.ncbi.nlm.nih.gov/projects/geo/).

### Statistical analysis

The characteristics of the patients who did and did not develop new distant metastasis until progressive disease (PD) were compared using Welch’s *t*-test [11] for continuous variables, Fisher’s exact test for two categorical variables, and Pearson’s χ^2^ test for more than two categorical variables. The prediction models based on serum miRNA levels were constructed using the logistic least absolute shrinkage and selection operator (LASSO) regression analysis with 10-fold cross-validation. The sensitivity, specificity, accuracy and area under the receiver operating characteristics (ROC) curve (AUC) were calculated for each model. To avoid overfitting, the optimal number of miRNAs in the model was determined based on the minimum cross-validated error. The association between the selected miRNAs and overall survival was analysed using the univariable Cox regression model. To adjust clinical variables that were marginally associated with overall survival (p < 0.1) in univariable analyses, multivariable Cox regression analysis was also performed.

Logistic LASSO regression analyses were performed with the software package R version 3.1.2 (R Foundation for Statistical Computing, http://www.R-project.org), compute.es package version 0.2–4, glmnet package version 2.0–3, hash package version 2.2.6, MASS package version 7.3–45, mutoss package version 0.1–10 and pROC package version 1.8. Unsupervised clustering and heatmap generation using Euclidean distance in Ward’s method for linkage analysis were performed using Partek Genomics Suite 6.6. Principal component analysis (PCA) was also performed using Partek Genomics Suite 6.6. Pearson’s χ^2^ test, Student’s *t*-test, ROC curve generation, the Kaplan-Meier plot with log-rank test and Cox regression analysis were performed using SPSS Statistics Version 24 (IBM SPSS, Armonk, N.Y., USA). All analyses were two-sided, and a p-value <0.05 was considered significant.

### Ethical concerns

This research was approved by the NCCH Institutional Review Board (2016–120, 2015–376). Written informed consent was obtained from each patient.

## Results

### Patient characteristics

In a median follow-up time of 117 months, all patients experienced progressive disease. A total of 147 patients, 52 patients developed at least one new distant metastasis at progression after eribulin monotherapy, whereas 95 patients did not develop new distant metastases. The patient characteristics of the two groups are compared in Table 1. Baseline patient characteristics and the characteristics of the breast cancer and treatment line did not differ significantly between the two groups, including biomarkers such as hormone receptors and HER2, the distribution of breast cancer, the treatment line of eribulin and the objective response to eribulin (Table 1).

**Table 1:** Patient characteristics.

		New distant metastasis				
Characteristic		Positive	(n = 52)	Negative	(n = 95)	P-value
Age—median [range]		54	[32–76]	59	[33–78]	0.066^a^
Follow-up period—median [range] (month)		8.6	[0.2–59.0]	12.5	[0.9–56.0]	
ER—no. (%)						0.442^b^
	+	35	(67.3)	70	(74.5)	
	-	17	(32.7)	24	(25.5)	
PgR—no. (%)						0.482^b^
	+	30	(57.7)	60	(63.8)	
	-	22	(42.3)	34	(36.6)	
HER2—no. (%)						0.810^b^
	+	8	(15.4)	13	(14.0)	
	-	44	(84.6)	80	(86.0)	
TNBC—no. (%)						0.500^b^
	+	11	(21.2)	15	(16.0)	
	-	41	(78.8)	79	(84.0)	
Stage—no. (%)						0.267^b^
	Recurrence	44	(84.6)	87	(91.6)	
	Stage IV	8	(15.4)	8	(8.4)	
ECOG PS–no. (%)						0.548^c^
	0	27	(51.9)	57	(60.0)	
	1	22	(42.3)	35	(36.8)	
	2	3	(5.8)	3	(3.2)	
No. of metastatic sites—no. (%)						0.861^b^
	≥3	22	(42.3)	38	(40.0)	
	<3	30	(57.7)	57	(60.0)	
Visceral disease—no. (%)						0.599^b^
	+	45	(86.5)	85	(89.5)	
	-	7	(13.5)	10	(10.5)	
Treatment line of eribulin—no. (%)						0.746^c^
	1	5	(9.6)	5	(5.3)	
	2	12	(23.1)	19	(20.0)	
	3	17	(32.7)	29	(30.5)	
	4	10	(19.2)	22	(23.2)	
	≥5	8	(15.4)	20	(21.0)	
Objective response to eribulin—no. (%)						0.369^c^
	PR	3	(5.8)	8	(8.4)	
	SD	26	(50.0)	54	(56.8)	
	PD	22	(42.3)	28	(29.5)	
	nonCR/nonPD	1	(1.9)	5	(5.3)	

a. Welch’s *t*-test

b. Fisher’s exact test

c. Pearson’s χ^2^ test

### Establishment of a prediction model for new distant metastases using a combination of miRNAs

**Fig 1:**
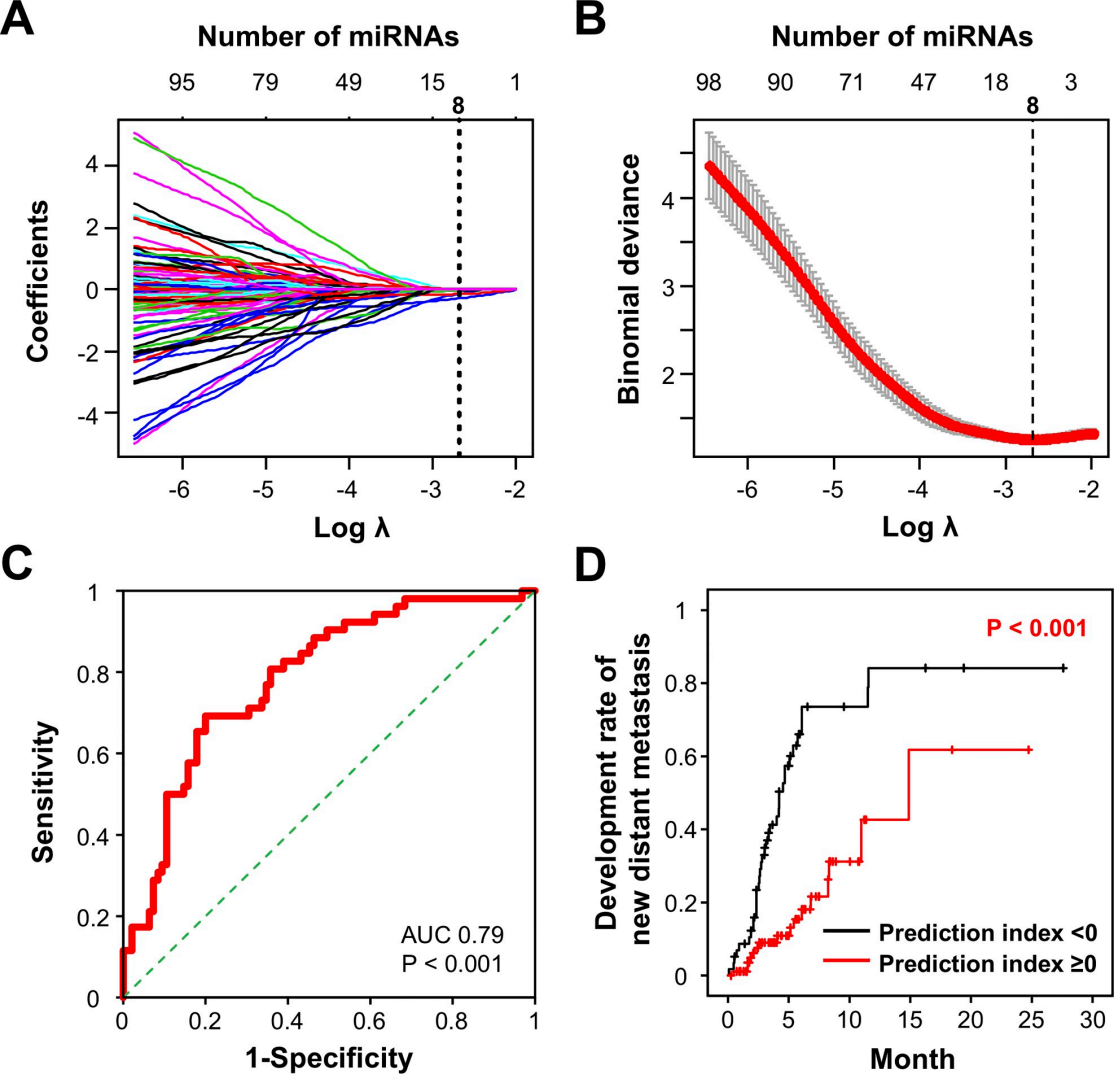
Establishment of prognostic biomarkers of responsiveness to eribulin in metastatic breast cancer patients. **A.** Coefficient path for the L1-regularised logistic regression applied to the cancer data plotted versus the logarithm of lambda (regularisation coefficient of L1 norm of the coefficient vector), relative to the norm of the estimate coefficients. The number of non-zero coefficients is shown above each plot. **B.** Cross-validation binomial deviance curve for logistic LASSO on the cancer data, with one-standard-error bands computed from 10-fold realisations. The vertical line on the left corresponds to the minimising value for logarithm Lambda. **C.** ROC curve analysis of the eight-miRNA combination predicting responsiveness to eribulin. AUC and p-values are shown in the plots. **D.** Kaplan-Meier plot of the rate of development of new distant metastases based on the prediction index. Black line: prediction index <0; Red line: prediction index ≥0.

Cross-validation by logistic LASSO regression analysis was performed to identify a combination of miRNAs for the prediction of eribulin responsiveness in metastatic breast cancer. To minimise overfitting in our model, the path coefficient and cross-validation binomial deviance curve for logistic LASSO were examined, and the optimal number of miRNAs in combination (n = 8) was determined based on the minimum cross-validated error (Fig 1A and 1B). The logistic LASSO regression model was used to identify prediction biomarkers by testing different combinations of one to eight miRNAs that could distinguish patients with newly developed distant metastases after eribulin treatment (Table 2). The analysis revealed that a combination of eight miRNAs (miR-4483, miR-8089, miR-4755-3p, miR-296-3p, miR-575, miR-4710, miR-5698 and miR-3160-5p) was optimal for predicting the appearance of new distant metastases with the highest accuracy. The prediction index was calculated using the following formula: exp Y / (1 + exp Y) -0.3731| Y = -0.01801×miR-4483–0.14291×miR-8089–0.01134×miR-4755-3p -0.14277×miR-296-3p -0.28373×miR-575–0.03178×miR-4710–0.01287×miR-5698–0.13961×miR-3160-5p + 4.80275. The ROC curve of the eight-miRNA combination showed the highest AUC of 0.79, with sensitivity of 0.69 and specificity of 0.76 (Fig 1C). Based on the prediction index, survival analysis was performed using the Kaplan-Meier method and log-rank test to compare overall survival after initiation of eribulin between patients with a high and low prediction index. Patients with a prediction index of <0 showed a significantly longer survival period than those with a prediction index of ≥0 (Fig 1D). Our model predicted the responsiveness to eribulin, which is tightly associated with the development of new distant metastases in metastatic breast cancer patients.

**Table 2:** Construction of prediction models using miRNAs.

Number of miRNAs	Sensitivity	Specificity	Accuracy	AUC
1^a^	0.92	0.27	0.50	0.62
2^b^	0.79	0.53	0.62	0.72
3^c^	0.77	0.60	0.66	0.74
5^d^	0.83	0.62	0.69	0.76
6^e^	0.69	0.76	0.73	0.77
7^f^	0.69	0.77	0.74	0.78
8^g^	0.69	0.82	0.76	0.79

a. (-0.0239)*miR-575–0.4624.

b. (-0.04785)*miR-575+(-0.01639)*miR-3160-5p -0.18945.

c. (-0.004077)*miR-296-3p+(-0.09225)*miR-575+(-0.050573)*miR-3160-5p +0.377653.

d. (-0.004538)*miR-8089+(-0.090765)*miR-296-3p+(-0.184948)*miR-575+(-0.00378)*miR-4710+(-0.106025)*miR-3160-5p +2.096777.

e. (-0.056646)*miR-8089+(-0.002592)*miR-4755-3p+(-0.115385)*miR-296-3p+(-0.220128)*miR-575+(-0.015284)*miR-4710+(-0.11774)*miR-3160-5p +3.061642.

f. (-0.002883)*miR-4483+(-0.079497)*miR-8089+(-0.005242)*miR-4755-3p+(-0.12544)*miR-296-3p+(-0.237057)*miR-575+(-0.020395)*miR-4710+(-0.122488)*miR-3160-5p +3.51243.

g. (-0.01801)*miR-4483+(-0.14291)*miR-8089+(-0.01134)*miR-4755-3p+(-0.14277)*miR-296-3p+(-0.28373)*miR-575+(-0.03178)*miR-4710+(-0.01287)*miR-5698+(-0.13961)*miR-3160-5p +4.80275.

### Diagnostic performance of single miRNAs for predicting the responsiveness to eribulin

**Fig 2:**
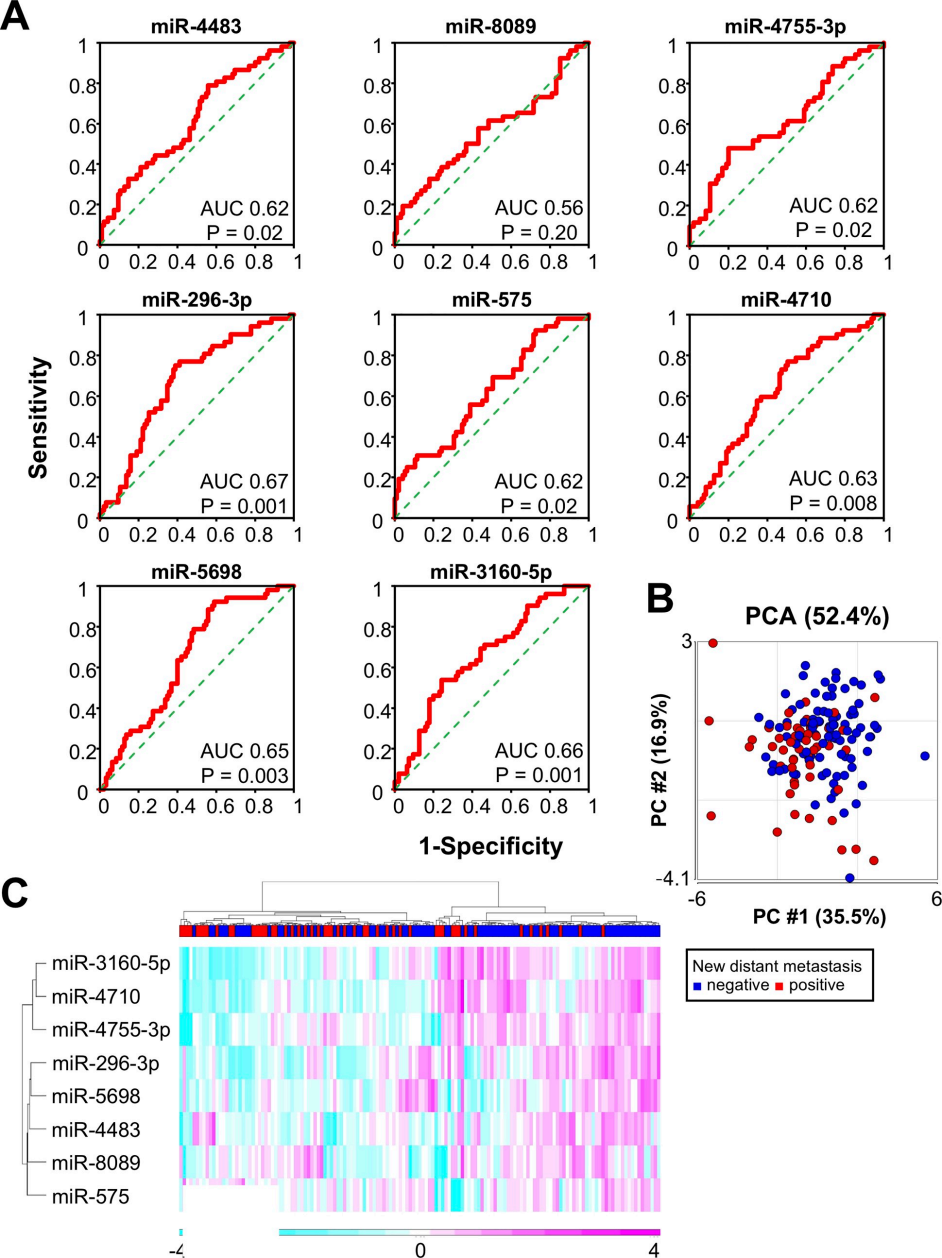
Eight selected miRNAs for the prediction of eribulin responsiveness in metastatic breast cancer. **A.** ROC curve analysis of the eight individual miRNAs. AUC and p-values are shown in the plots. **B.** PCA map of 53 new metastasis-positive samples (red) and 96 new metastasis-negative samples (blue) in metastatic breast cancer patients. **C.** Unsupervised hierarchical clustering analysis with a heatmap showing 149 metastatic breast cancer samples prior to eribulin treatment with eight selected miRNAs.

Eight selected miRNA profiles from our experimental cohort were examined. Seven of the eight miRNAs were statistically effective in distinguishing metastatic breast cancer patients who developed new distant metastases after eribulin treatment (AUC: 0.62–0.67, p < 0.05, Fig 2A). PCA of the eight selected miRNAs suggested a rough separation of metastasis-positive and -negative groups (Fig 2B). Unsupervised hierarchical clustering using a heatmap with the eight selected miRNAs in all samples also suggested rough segregation of metastasis-positive and -negative groups (Fig 2C).

The Cox regression analysis was performed to determine overall survival according to the expression of each miRNA and clinicopathological characteristics (Table 3). The results of multivariable analysis indicated that lower expression levels of miR-8089 (hazard ratio [HR], 0.61; 95% confidence interval [CI], 0.37–0.98) and miR-5698 (HR, 0.76; 95% CI, 0.62–0.92) were significantly associated with improved overall survival, as well as age, ECOG performance status and number of metastatic sites.

**Table 3:** Cox regression analysis of overall survival.

	Univariable			Multivariable^a^		
	HR	(95% CI)	P-value	HR	(95% CI)	P-value
miR-4483 (per 2 times)	1.02	(0.84–1.23)	0.840			
miR-8089 (per 2 times)	0.52	(0.33–0.8)	**0.003**	0.61	(0.37–0.98)	**0.045**
miR-4755-3p (per 2 times)	1.01	(0.88–1.15)	0.848			
miR-296-3p (per 2 times)	1.00	(0.74–1.34)	0.993			
miR-575 (per 2 times)	0.88	(0.72–1.06)	0.185			
miR-4710 (per 2 times)	0.89	(0.75–1.06)	0.203			
miR-5698 (per 2 times)	0.74	(0.61–0.9)	**0.003**	0.76	(0.62–0.92)	**0.008**
miR-3160-5p (per 2 times)	0.97	(0.82–1.13)	0.712			
Age (per 10 yr)	0.70	(0.56–0.87)	**0.002**	0.71	(0.56–0.88)	**0.002**
ER positive	0.93	(0.57–1.5)	0.760			
PgR positive	0.78	(0.5–1.19)	0.254			
HER2 positive	0.65	(0.32–1.29)	0.218			
TNBC positive	1.71	(0.98–2.95)	0.057	1.60	(0.88–2.9)	0.119
Stage IV	0.93	(0.44–1.93)	0.847			
ECOG PS ≥1	1.76	(1.14–2.68)	**0.010**	1.91	(1.22–2.98)	**0.004**
No. of metastatic sites ≥3	1.62	(1.04–2.48)	**0.029**	1.62	(1.01–2.58)	**0.043**
Presence of visceral disease	1.00	(0.54–1.83)	0.988			

a. adjusted for marginally associated factors (p < 0.1) in univariable analyses.

HR, hazard ratio; CI, confidence interval.

## Discussion

Eribulin has both cytotoxic and non-cytotoxic mechanisms of action. Some reports show that eribulin exerts complex non-cytotoxic effects on the tumour microenvironment such as on surrounding stromal cells[12–14]^4^. Eribulin treatment reversed the epithelial-mesenchymal transition (EMT) phenotype, and pretreatment of surviving triple-negative breast cancer (TNBC) cells *in vitro* with eribulin for 7 days decreased the number of lung metastases in an *in vivo* experimental metastasis model [14, 15]. The ratio of tumour-infiltrating lymphocytes in TNBC was identified as a predictive factor of responsiveness to eribulin [16]. Although these observations provide insights into the mechanisms underlying eribulin activity, they remain to be validated in a clinical setting, and identification of factors that predict responsiveness to eribulin in a clinical setting would be highly desirable. In the present study, serum miRNAs from 147 metastatic breast cancer patients were analysed, including 52 cases with new distant metastasis and 95 cases without new distant metastasis after eribulin treatment. From all samples, comprehensive profiles of 2565 miRNAs were obtained using a highly sensitive miRNA microarray analysis on a standardised platform (3D-Gene^®^, Toray Industries, Inc.). Based on the cross-validation by logistic LASSO regression analysis, we established a prediction model of the responsiveness to eribulin in metastatic breast cancer based on an eight-miRNA combination. In this prediction model, ROC curve analysis estimated the probability of newly developed distant metastasis with an AUC of 0.79, a sensitivity of 0.69 and a specificity of 0.82. In addition, high expression of two of the eight selected miRNAs (miR-8089 and miR-5698) was significantly correlated with poor survival in metastatic breast cancer patients by Cox regression analysis.

Baseline biomarkers of serum miRNA levels are not only useful in predicating prognosis in cancer patients [17], but also help predict drug responses, allowing better treatment choices for patients. Some studies have reported a relationship between serum miRNAs and drug responsiveness in cancer. For instance, five miRNA signatures (miR-224, miR-455-3p, miR-1236, miR-33a, and miR-520d-3p) can predict responsiveness to R-CHOP treatment (rituximab, cyclophosphamide, Adriamycin, vincristine, and prednisone) in diffuse large B cell lymphoma [18]. ROC analysis revealed AUC values of 0.63–0.79 for each of these miRNAs, and the predictive accuracy of responsiveness to eribulin of the serum miRNAs identified in this study was similar (AUC: 0.79). Another study showed that baseline serum miR-125 levels in non-responders to neoadjuvant chemotherapy were higher than those in responders among stage II/III breast cancer patients, and serum miR-21 levels in responders were significantly decreased after neoadjuvant chemotherapy [19]. In addition to miRNA profiling in patient serum, baseline factors in peripheral blood are also considered predictors of responsiveness to anti-cancer drugs such as immune checkpoint inhibitors. In other studies, peripheral blood factors, such as low serum lactate dehydrogenase and relative lymphocyte count, were shown to be associated with better outcomes for patients with melanoma treated with pembrolizumab or ipilimumab, a PD-1 monoclonal antibody and a CTLA4 monoclonal antibody, respectively [20, 21]. In addition to its use in predicting cancer prognosis, serum miRNA profiling has been employed to predict drug responsiveness in other diseases. For instance, the expression levels of several serum miRNAs predicted responses to TNFα inhibitors (miR-99a and miR-143 for adalimumab; miR-23a and miR-197 for etanercept) in rheumatoid arthritis [22]. Although how the miRNAs identified in this study function in mediating responsiveness to eribulin is unknown, this kind of approach could be useful in predicting a prognosis or responsiveness to any type of anti-cancer drug.

The present study had several limitations, including the lack of a validation cohort. The serum samples used to analyse miRNAs were collected only at the NCCH; therefore, the sample size was not statistically large enough to divide into training and validation cohorts. Despite cross-validation by logistic LASSO regression analysis, further investigations using an external validation cohort are necessary. Another limitation of the study is the lack of a functional assay for the selected miRNAs. The present data showed a correlation between high expression levels of miR-8089 and miR-5698 and poor survival, suggesting the oncogenic roles of these miRNAs. However, many circulating miRNAs related to cancer types are not disease specific and likely originate from blood cells [23]. Furthermore, functional analysis of these miRNAs is still lacking, and how they affect responsiveness to eribulin in breast cancer cells or animal models remains unknown. Additionally, based on the literature, miR-296-3p and miR-575 are associated with cancer progression [24–27]. The association of these miRNAs with responsiveness to eribulin and the development of distant metastases in metastatic breast cancer remains unclear.

## Conclusions

In conclusion, to the best of our knowledge, this is the first report showing that circulating miRNA profiles before eribulin treatment can predict the future emergence of new distant metastases. We established a blood-based less invasive test for the prediction of eribulin responsiveness in metastatic breast cancer patients by identifying an eight-miRNA combination. This prediction model can also serve as a prognostic or predictive factor for overall survival after the start of eribulin treatment. This result indicated that the baseline conditions may differ between responders and non-responders to eribulin. Based on an analysis of a limited number of samples, the AUC of the eight-miRNAs for prediction of responsiveness to eribulin was only 0.79 while the sensitivity in only 0.69. Further research will be required to confirm this result using a largest number of samples.
